# Case Report: A rare and recurrent case of primary corneal keratosis: diagnostic and therapeutic challenges

**DOI:** 10.3389/fmed.2025.1678333

**Published:** 2026-01-20

**Authors:** Lihong Huang, Dazheng Zhang

**Affiliations:** 1 Dujiangyan Medical Centre, Chengdu, China; 2 Chengdu University of Traditional Chinese Medicine, Chengdu, China; 3 Dujiangyan Juvenile Myopia Prevention and Treatment Center, Chengdu, China

**Keywords:** cutaneous horn, cornea mass, recurrent proliferation, solar keratosis, therapeutic challenges

## Abstract

**Background:**

Ocular surface keratosis typically presents as a focal, hyperkeratotic lesion. While commonly associated with actinic damage on the eyelid margin or conjunctiva, primary corneal involvement is exceptionally rare.

**Case presentation:**

A 32-year-old Asian female presented with a 10-year history of a recurrent white mass on the right cornea. Slit-lamp examination revealed a 3 × 3 × 4 mm, irregular, broad-based keratotic lesion at the nasal limbus, extending 1 mm onto the clear cornea. Anterior segment optical coherence tomography (OCT) confirmed superficial stromal infiltration. The patient had undergone prior surgical excision at an external hospital, followed by multiple recurrences. At our institution, conservative management with repeated mechanical debridement under slit-lamp guidance was performed. Histopathological analysis of the debrided material showed compact hyperkeratosis without cellular atypia, consistent with a benign keratotic lesion. Despite these interventions, the lesion demonstrated a consistent and rapid recurrence pattern, regrowing within 2–3 weeks after each debridement over a 12-month follow-up period.

**Conclusion:**

This case describes a rare presentation of primary recurrent corneal keratosis with benign histopathological features but clinically aggressive behavior. The rapid and persistent recurrence despite conservative management highlights the limitations of superficial debridement and suggests the presence of subclinical disease within the lesion base. This report underscores the need for long-term surveillance and consideration of more definitive treatment strategies for similar recurrent keratotic lesions on the cornea.

## 1 Introduction

Focal keratosis of the ocular surface (1–3), encompassing the conjunctiva and cornea, represents a clinical sign of abnormal keratinization rather than a definitive pathological diagnosis. These lesions can arise from a spectrum of underlying conditions, ranging from benign idiopathic keratosis to premalignant actinic keratosis and malignant squamous cell carcinoma. Their clinical significance lies in the need to exclude malignancy and their potential for recurrence, which can lead to persistent symptoms, cosmetic concerns, and in corneal cases, visual impairment or surface irregularity.

While keratotic lesions are well-documented on the eyelid skin and conjunctiva, primary involvement of the cornea remains exceptionally rare (4). The cornea is a non-keratinized, stratified squamous epithelium, and the development of a significant keratotic plaque is a pathological deviation. This anatomic distinction makes corneal keratosis a unique clinical entity. Most literature on corneal “horns” or keratosis consists of isolated case reports, and the natural history and optimal management of recurrent cases are not well-established due to their scarcity.

The disease was officially recognized as a medical disorder in the late eighteenth century by London surgeons Everard Home and his brother-in-law John Hunter, thereby establishing its characterization within the realm of medicine (5). To our knowledge, this represents the first documented case of primary corneal keratosis displaying such a persistent and rapid recurrence pattern despite benign histology. A comprehensive literature search conducted in PubMed, Embase, and Web of Science using the terms “corneal keratosis,” “corneal cutaneous horn,” “primary corneal keratosis,” and “recurrent corneal keratosis” from inception to 2024 revealed no previously reported cases of primary corneal keratosis with a documented history of rapid, persistent recurrence over a decade, despite multiple interventions and benign histopathology. We report the case of cornea cutaneous horn in a 32-year-old Asian female patient presenting with corneal mass. The ethical approval for this case report was granted by the Ethics Committee of Dujiangyan People's Hospital, with the approval number: 2023-S-28.

## 2 Case presentation

The patient's clinical course involved multiple interventions across two phases. She reported a 10-year history of a gradually enlarging corneal mass prior to its initial diagnosis and surgical intervention at an external hospital. Prior to presentation at our institution, the patient reported undergoing surgical excision of the corneal mass at an external hospital, described as a “local conjunctival and superficial scleral lesion excision.”

However, critical details (e.g., surgical technique, histopathology) remain unverified due to lost medical records, relying solely on the patient's account. Following referral to our clinic, the patient declined further surgical intervention citing concerns over risks and the absence of symptoms (e.g., pain, vision loss). Conservative management was adopted, consisting of repeated outpatient mechanical debridement under slit-lamp guidance (February and October 2023, with subsequent follow-ups), where superficial keratinized material was removed using sterile cotton swabs without adjuvant therapies. Despite transient relief, these procedures failed to address deeper keratinized tissue, leading to persistent recurrence.

### 2.1 Initial presentation and clinical findings

A 32-year-old Asian female presented to our ophthalmology clinic with a chief complaint of a recurrent white mass on the right eye. The lesion was first noted a decade prior and had been slowly enlarging. She reported undergoing an excisional procedure at an external hospital 1 year earlier, but the mass recurred within 1 month. No records from that prior intervention were available.

**Figure 1:**
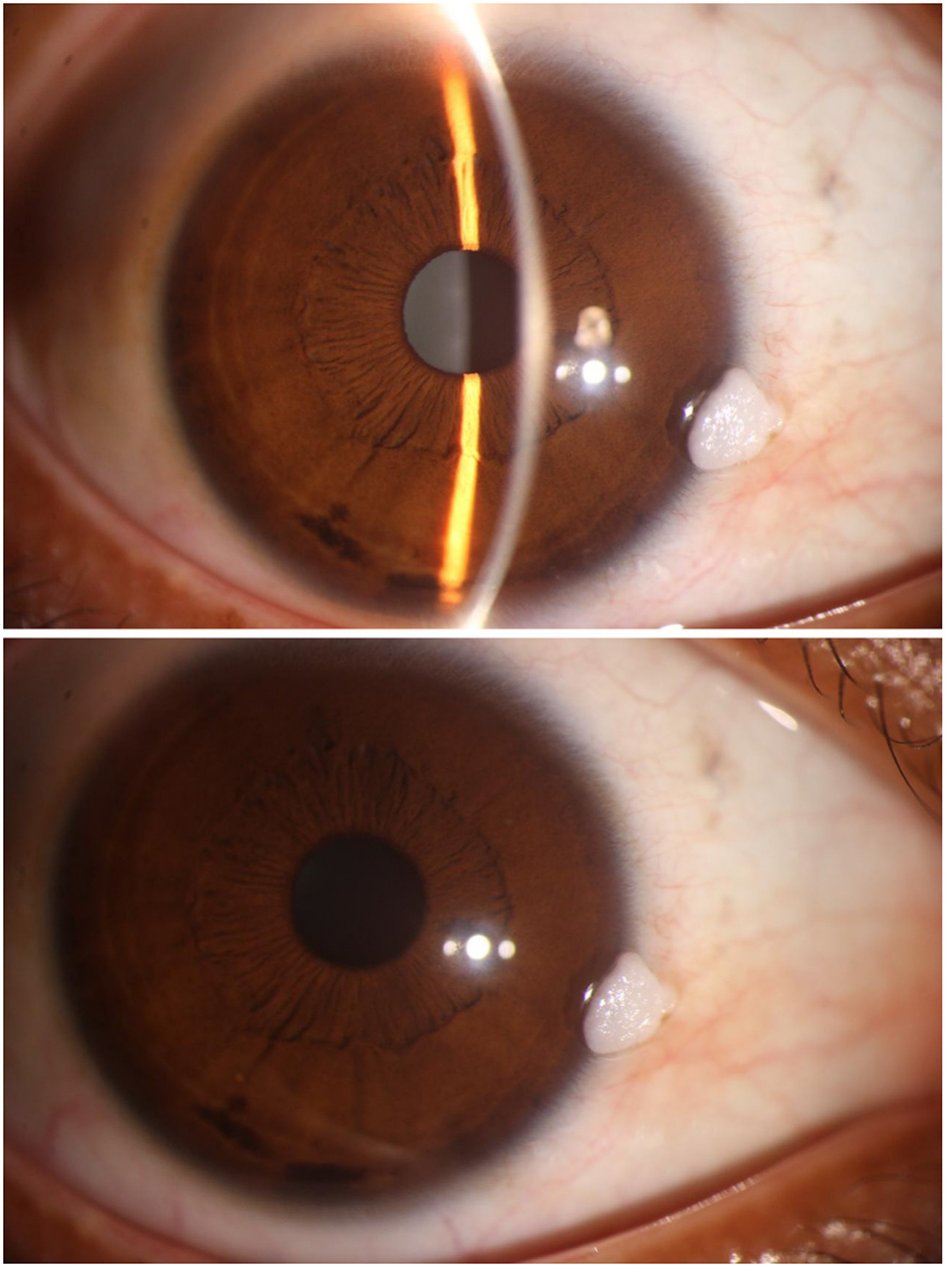
Mass surface-Initial examination with slit-lamp. An irregular, white, broad-based mass (3 × 3 × 4 mm) is observed on the nasal conjunctiva, extending 1 mm into the clear corneal margin (between arrows). The lesion exhibits a friable, hyperkeratotic surface with well-defined borders. Note the absence of conjunctival congestion or neovascularization.

On examination, her best-corrected visual acuity was 1.0 in both eyes. Slit-lamp examination of the right eye revealed a 3 × 3 × 4 mm, irregular, sessile, and white hyperkeratotic mass. The lesion was broad-based at the nasal limbus, with its epicenter on the conjunctiva and a 1 mm extension onto the clear cornea (Figure 1). The surface was friable, with well-defined borders and no associated conjunctival injection or neovascularization. Corneal transparency outside the lesion and anterior chamber structures were normal.

The patient denied any familial history of similar ocular lesions, keratinization disorders, or genetic skin conditions.

### 2.2 Diagnostic evaluation

**Figure 2:**
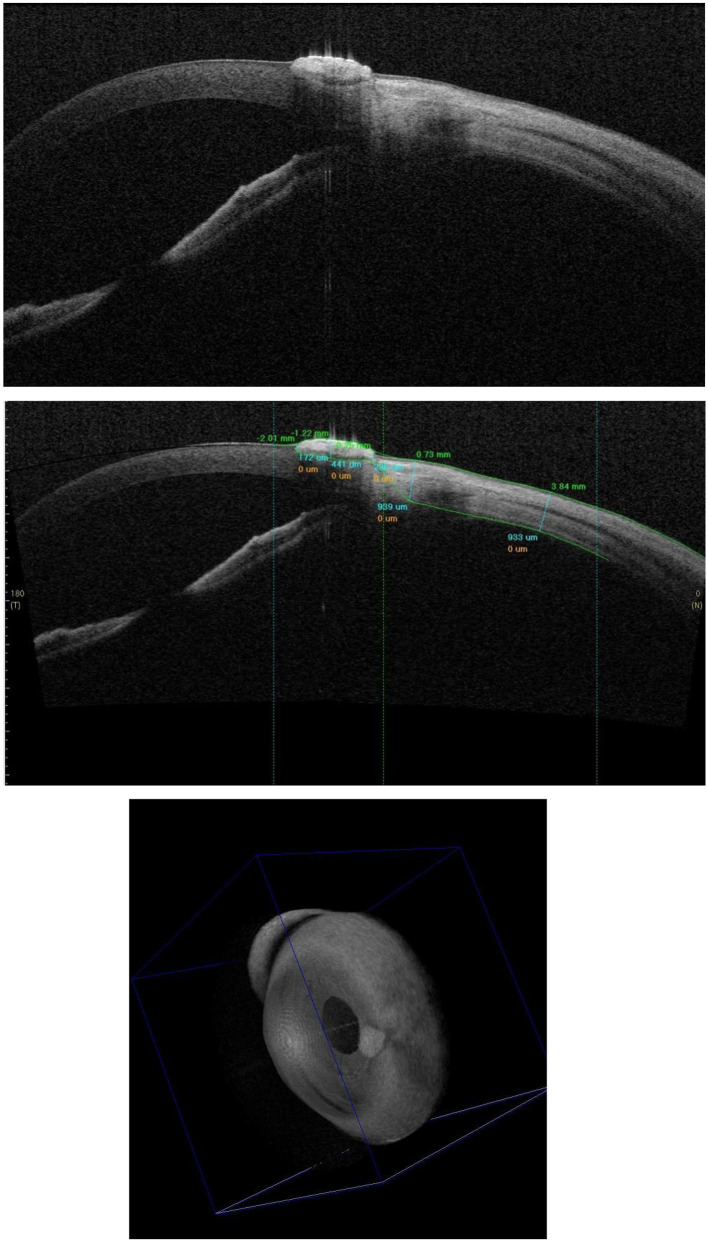
Mass surface: Initial OCT examination. The cross-sectional image confirms stromal infiltration by the hyperreflective mass (yellow arrow). The underlying corneal layers appear intact, with no evidence of deep invasion into the anterior chamber.

Anterior segment optical coherence tomography (AS-OCT) was performed, which clearly demonstrated a hyperreflective mass arising from the surface, causing disruption of the normal epithelial and anterior stromal architecture and confirming superficial stromal infiltration (Figure 2). The primary differential diagnoses based on clinical appearance and location included a benign keratotic lesion.

Our diagnostic approach was limited by the patient's strong aversion to any invasive procedure that could potentially scar or affect vision. Therefore, a full-thickness biopsy for definitive histopathological characterization was not feasible. The initial management plan was to perform a superficial debridement for both therapeutic and diagnostic purposes, allowing for histopathological analysis of the exophytic component.

### 2.3 Interventions and histopathology

**Figure 3:**
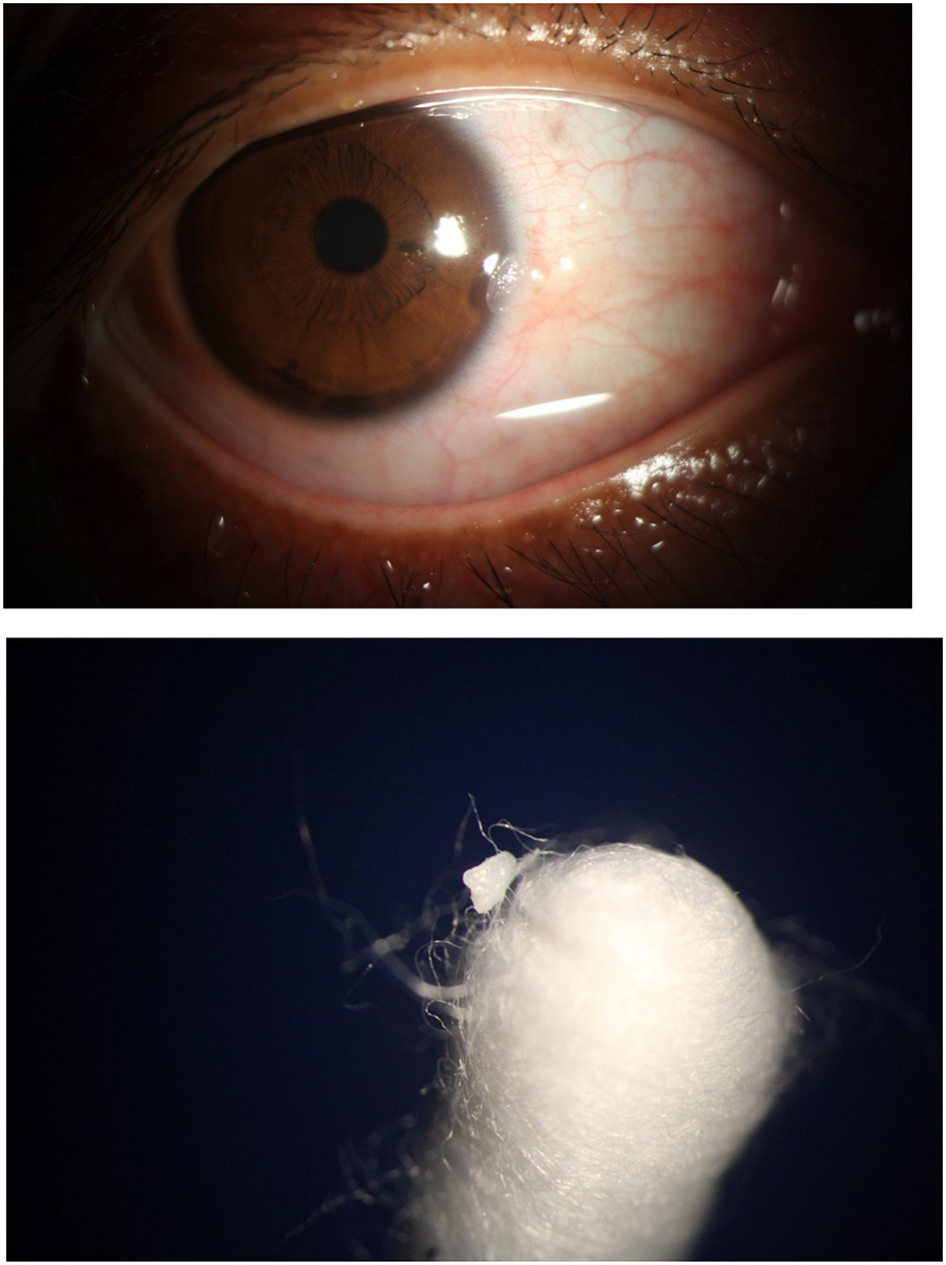
Slit-lamp photograph immediately post-swabbing (October 2023). Following gentle debridement of superficial keratinized debris, the fragile lesion base is exposed (white arrow), showing a smooth, non-ulcerated surface without active bleeding.

In October 2023, under slit-lamp guidance, superficial mechanical debridement of the keratinized material was performed using a sterile cotton swab. The underlying base appeared smooth and non-ulcerated after the procedure (Figure 3).

**Figure 4:**
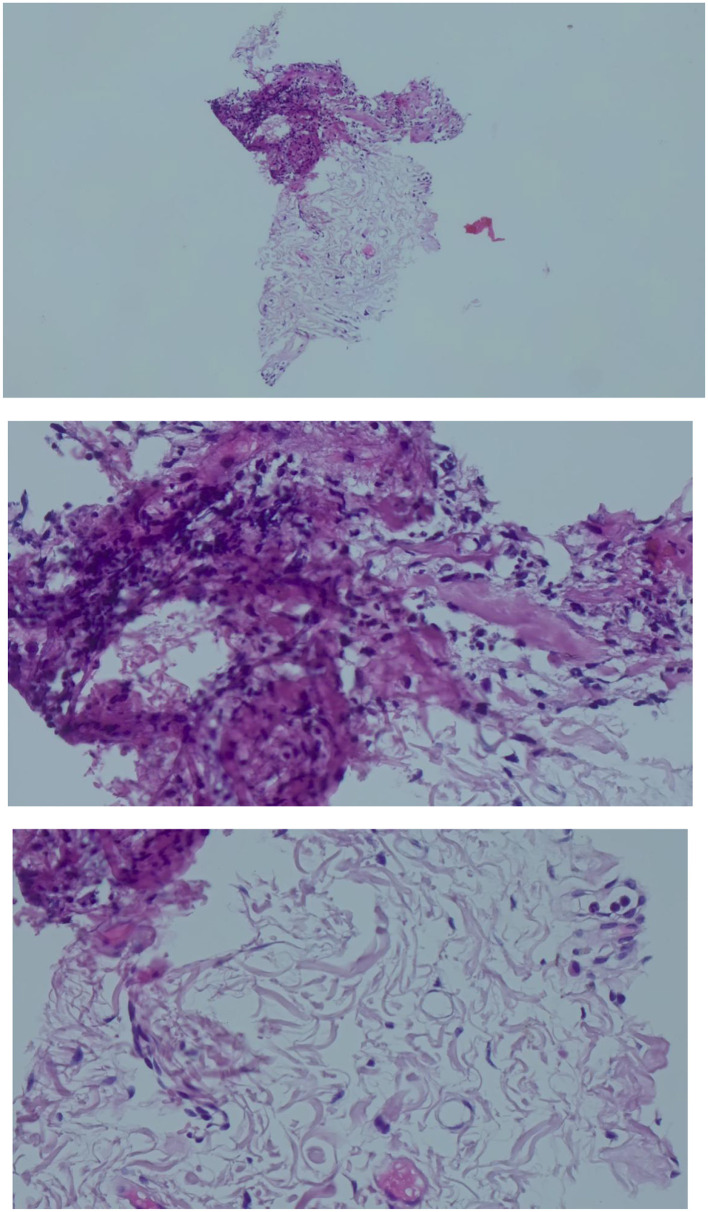
Histopathological examination of the debrided material (H&E staining). Photomicrograph reveals concentric layers of keratinized epithelial cells (red bracket), consistent with a cutaneous horn. Key findings include compact hyperkeratosis and the absence of cytological atypia or malignant features, ruling out underlying carcinoma at this stage.

The debrided material was sent for histopathological examination. Hematoxylin and eosin staining revealed a markedly thickened layer of compact, hyperkeratotic stratum corneum, with an underlying attenuated granular layer. The underlying squamous epithelium showed regular maturation without cytological atypia, basal cell hyperplasia, or mitotic figures. There was no evidence of viral cytopathic changes or dysplasia. The subepithelial stroma was devoid of a significant inflammatory infiltrate (Figure 4). These findings were consistent with a benign, hyperkeratotic lesion.

### 2.4 Clinical course, follow-up, and recurrence

Given the benign histology and the patient's preference to avoid surgical risks, a conservative management strategy was adopted. This involved “watchful waiting” and planned debridement only for symptomatic relief or significant cosmetic concern, forgoing adjuvant therapies like cryotherapy or topical chemotherapeutic agents.

**Figure 5:**
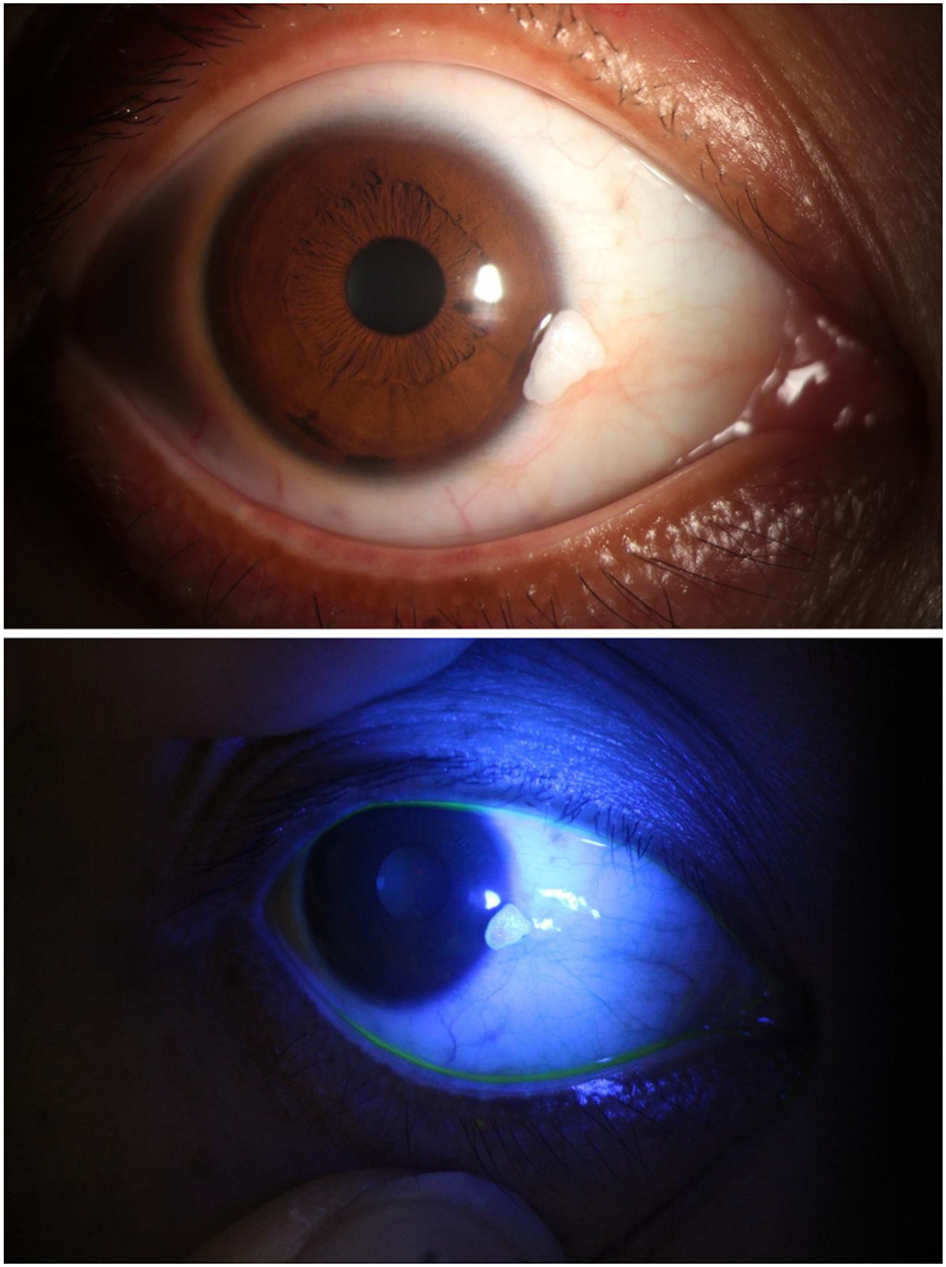
First documented recurrence (18 days post-swabbing, November 2023). Rapid regrowth of the keratinized lesion is observed at the original site (white arrow), demonstrating an identical morphological appearance to the initial presentation, which suggests residual pathological tissue rather than *de novo* formation.

**Figure 6:**
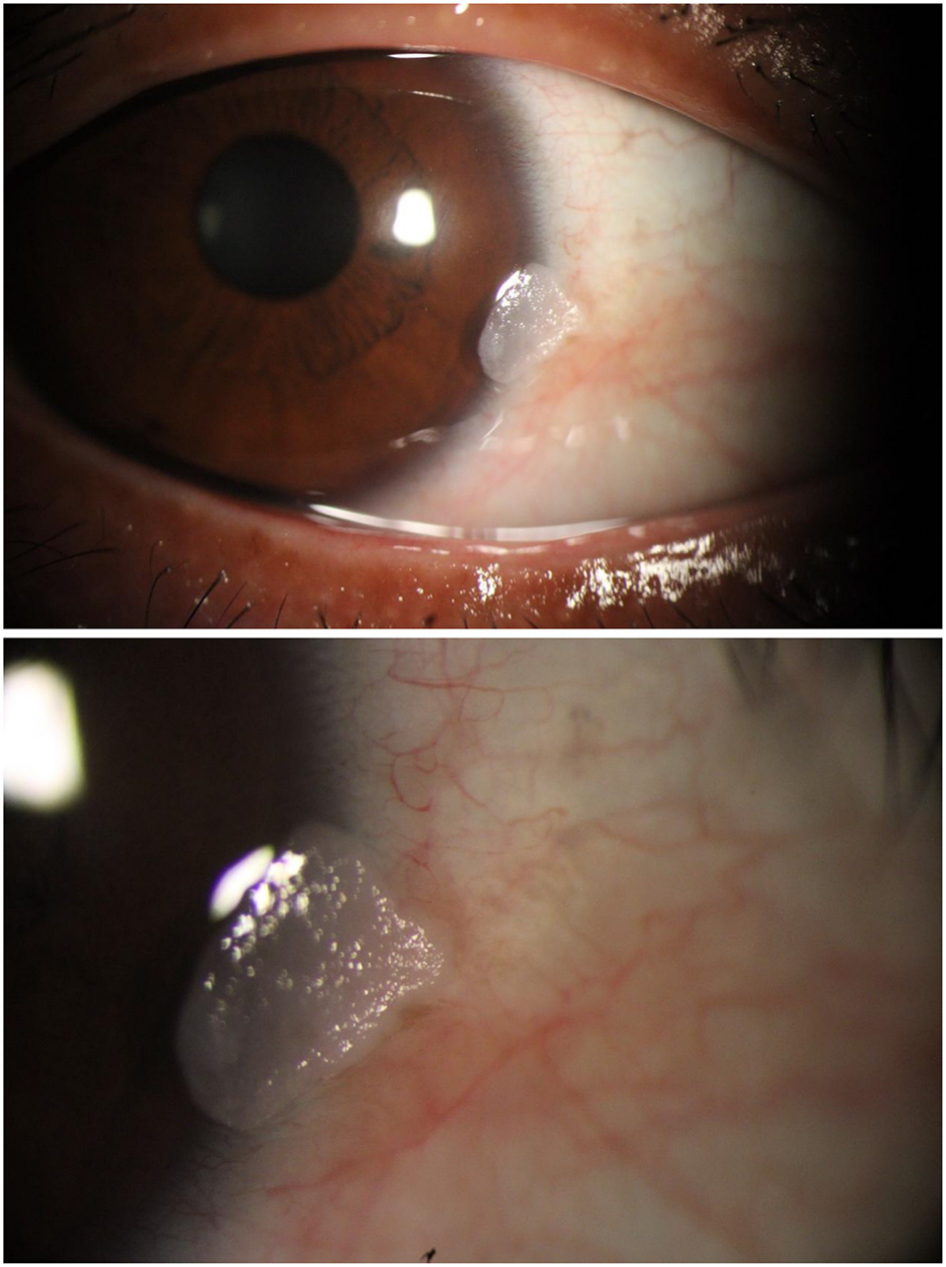
Slit-lamp photograph at 18-day follow-up (December 2023). The lesion shows persistent regrowth (white arrow) with a friable, white, hyperkeratotic surface, consistent with the previous recurrence pattern aobserved after conservative management.

**Figure 7:**
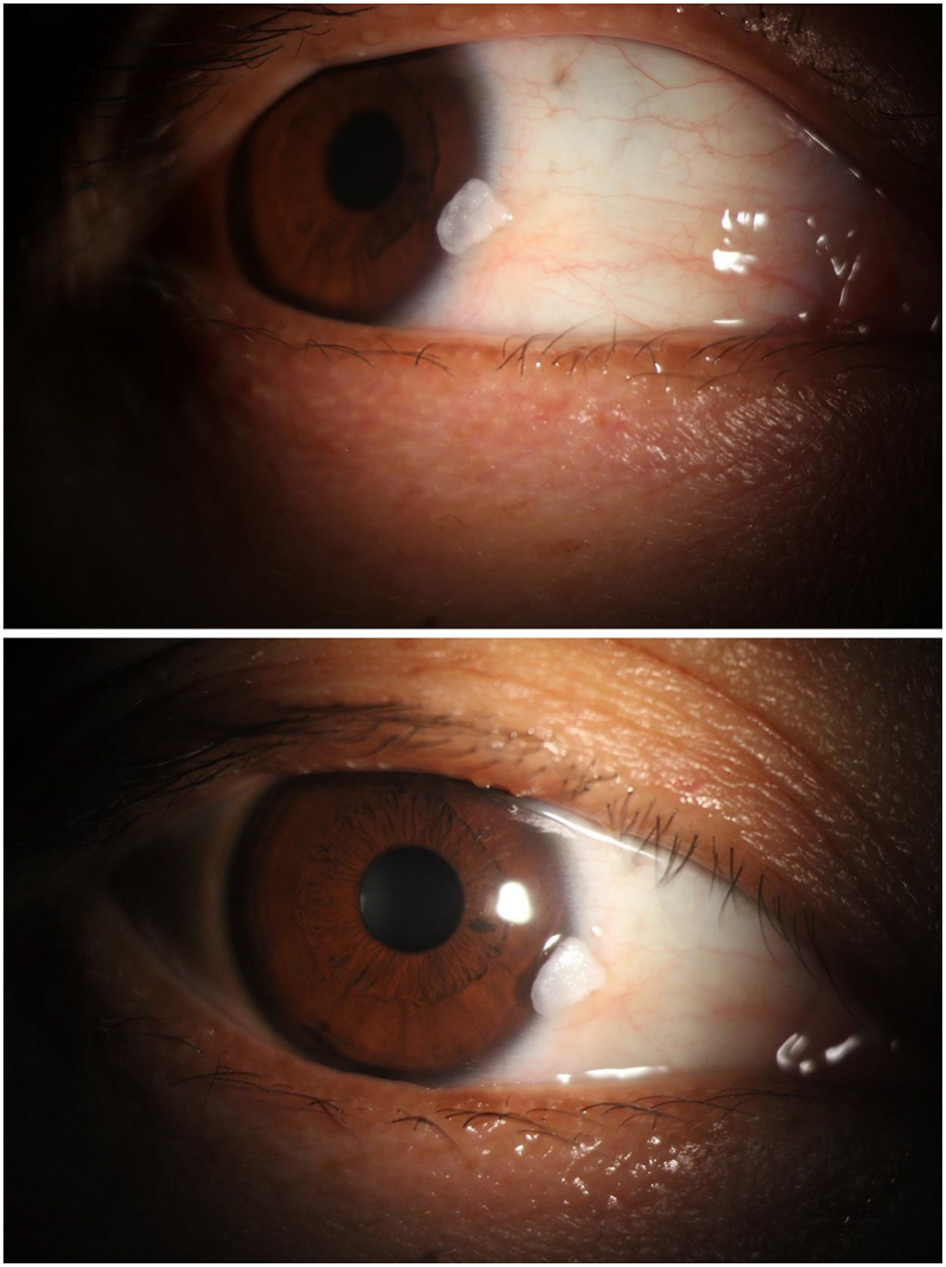
Slit-lamp photograph at 1-month follow-up. The recurrent mass (white arrow) maintains stable dimensions and characteristic morphology, highlighting the cyclical nature of regrowth despite repeated swabbing procedures.

The clinical course over the subsequent year was characterized by persistent recurrence. The lesion consistently regrew to its original size within 2–3 weeks after each debridement. The timeline and pattern of recurrence are summarized in Table 1. The regrowth was morphologically identical each time (Figures 5–7), suggesting regeneration from a persistent pathological base rather than *de novo* formation. The patient expressed dissatisfaction with the need for repeated procedures but remained firm in her refusal of more definitive, invasive excision. Throughout the follow-up period, the lesion's dimensions remained stable, and no clinical signs of malignant transformation, such as ulceration or neovascularization, were observed.

**Table 1:** Follow-up timeline.

**Date**	**Event**	**Imaging/procedures**	**Patient decision**
Feb 2023	Initial excision at external hospital	No records available	–
Mar 2023	First recurrence	Slit-lamp partial excision	Declined further surgery
Oct 2023	Slit-lamp swabbing at our institution	OCT, histopathology (Figures 3–4)	Refused medical/surgical therapy
Nov 2023	Recurrence post-swabbing	Slit-lamp documentation (Figure 5)	Opted for repeat swabbing
Dec 2023	Persistent regrowth	Follow-up (Figure 6)	Continued refusal of invasive care

Over 12 months, the lesion demonstrated stable dimensions but persistent recurrence post-swabbing. Determination of complete removal during each procedure was based on slit-lamp criteria: absence of visible keratinized material, smooth conjunctival surface, and clear corneal margin. However, the fragility of the lesion base and the patient's preference for minimal intervention limited the depth of debridement, likely contributing to residual subclinical disease and subsequent recurrence. To differentiate between true recurrence and incomplete removal, we monitored the timeline and pattern of regrowth. Rapid reappearance (within 2–3 weeks) of histologically similar keratinized material at the identical site supported the likelihood of incomplete excision rather than *de novo* recurrence.

## 3 Discussion

### 3.1 Patient perspective

The patient reported no functional impairment (e.g., visual acuity preserved at 1.0) but expressed significant anxiety regarding the lesion's cosmetic appearance. This concern heavily influenced her preference for conservative management despite recurrent growth.

### 3.2 Diagnostic and management challenges

Cutaneous horns are rare tumors on the eyelid skin, accounting for approximately 4% of all eyelid lesions, and can vary greatly in size and shape (6, 7). CHs typically arise in sun-exposed regions such as the head, neck, and extremities, while lesions on the trunk or non-exposed limbs are uncommon (6) (Table 2). Prior reports of eyelid CHs (4) However, despite ongoing research, the exact pathogenesis of this keratinized abnormality remains incompletely understood (8).

**Table 2:** Differential diagnoses.

**Diagnosis**	**Key features**	**Ruling-out evidence in this case**	**References**
Actinic keratosis (AK)	Hyperkeratosis, solar elastosis, mild atypia	No solar elastosis or significant atypia on histopathology	Mencia-Gutierrez, et al. (4)
Squamous cell carcinoma (SCC)	Cytological atypia, stromal invasion, mitoses	Absence of atypia, invasion, or mitotic figures in serial biopsies	Yu, et al. (6)
HPV-related papilloma	Koilocytes, papillomatous architecture, HPV PCR+	Negative HPV PCR, no koilocytes or viral inclusions	Bondeson (8) Lee, et al. (18)
Seborrheic keratosis	“Stuck-on” appearance, pseudohorn cysts, melanin deposition	Lack of pseudohorn cysts/melanin; lesion location inconsistent with typical seborrheic keratosis	Schwartz (19)
Pseudoepitheliomatous hyperplasia	Reactive epidermal hyperplasia, inflammatory infiltrate	No underlying inflammation or trauma history; lesion behavior inconsistent with reactive hyperplasia	Requena, et al. (20)

The cornerstone of managing any keratotic lesion is a definitive histopathological diagnosis to exclude malignancy, typically achieved through complete excision. In this case, the corneal location and the patient's strong preference against invasive procedures created a unique constraint. Our diagnostic approach was consequently limited to superficial debridement, which, while confirming the absence of malignancy in the exophytic component, could not fully evaluate the underlying epithelial base from which the recurrence likely originated. This highlights a critical clinical dilemma: when standard diagnostic and therapeutic excision is not feasible, clinicians must rely on a combination of clinical monitoring, imaging, and limited biopsy, accepting the inherent diagnostic uncertainty.

The rationale for our conservative management was two-fold: first, the repeated histopathological findings were consistently benign, and second, we respected the patient's autonomy and her assessment of the risks and benefits. However, the failure of this approach to prevent recurrence demonstrates that for this particular entity, superficial debridement is merely palliative and does not address the root cause of the pathological keratinization.

### 3.3 Actinic keratosis potential

Actinic keratosis (AK) presents clinically as flat, slightly raised lesions with clear borders, appearing white or yellow-white, and is a non-invasive tumor typically affecting only one eye (7–10).

Histopathological analysis revealed hyperkeratosis and loss of the granular layer, overlapping with features of AK. However, the absence of cytological atypia precluded a definitive diagnosis of AK. We hypothesize that this lesion may represent an early or variant form of AK, necessitating long-term follow-up and adjunctive immunohistochemical studies (e.g., p53, Ki-67) to assess malignant potential (11). The occurrence of AK within the conjunctiva remains controversial, with potential associated factors including UV exposure, chronic inflammation, superficial trauma, and human papillomavirus (HPV) infection (12, 13). While UV exposure is a known risk factor for CHs, our patient denied significant outdoor activity. Alternative etiologies include chronic mechanical irritation (e.g., contact lens), genetic predisposition to keratinization disorders (e.g., KRT mutations), or subclinical HPV infection (14). Further investigation into these factors is warranted.

Histopathologically, AK is characterized by epithelial hyperplasia and hyperkeratosis of the conjunctival epithelium, loss of the granular layer, thickening of the spinous layer, basal cell hyperplasia, actinic elastosis, and infiltration of lymphocytes and plasma cells (15). Histologically, there is thickening of the epidermal stratum corneum with scattered areas of keratosis, loss of the granular layer, and epidermal thickening. These features are crucial for distinguishing AK from other tissue pathological conditions and contribute to a more comprehensive understanding in this case.

### 3.4 Clinical implications and future directions

The rapid, cyclical regrowth of histologically bland tissue suggests the presence of a persistent driver within the local limbal or corneal epithelial cells. While our study did not include molecular investigations, the observed clinical behavior points to several compelling questions for future research. Investigating potential mechanisms, such as the presence of subclinical epithelial dysplasia, localized stem cell dysfunction at the limbal base, or the role of chronic microtrauma could provide insights into the pathogenesis of recurrence. Techniques such as immunohistochemical staining for proliferation markers or tumor suppressor proteins on biopsy specimens, or *in vivo* confocal microscopy to visualize cellular-level changes, could be valuable tools in future cases to probe beneath a bland histological appearance.

Therefore, for similar recurrent cases, we recommend a more proactive diagnostic strategy. If patient consent can be obtained, a shallow but full-width biopsy of the lesion base after debridement could yield more informative tissue. Furthermore, based on the lessons learned from this case, a more definitive intervention, such as controlled superficial keratectomy with adjunctive cryotherapy or mitomycin C application, could be considered to eradicate the pathological niche and achieve better long-term control (16, 17).

### 3.5 Differential diagnoses

Considering that the patient strongly refused further examination, we will further discuss the relevant identification of HPV in this case. Further studies using hybridization capture sequencing or methylation profiling may clarify HPV's role in similar cases. The recurrent corneal lesion's clinical and histopathological features necessitated differentiation from several entities. Below is a comparative analysis of key differentials:

### 3.6 Alternative treatment strategies

Beyond the conservative management employed in this case, several alternative treatment modalities exist for CHs, particularly for lesions with aggressive recurrence or concerning cosmetic outcomes. Surgical excision remains the gold standard, offering the dual advantage of complete lesion removal and acquisition of a full-thickness tissue specimen for definitive histopathological diagnosis, which is crucial for ruling out underlying malignancy. For smaller or superficial lesions, ablative options such as cryotherapy with liquid nitrogen or carbon dioxide laser vaporization are effective alternatives, providing good cosmetic results with minimal bleeding and precise tissue destruction. Laser therapy, in particular, may be advantageous for periocular lesions due to its ability to contour to the delicate eyelid anatomy. Additionally, adjunctive pharmacological therapies can be considered, especially when actinic keratosis is suspected or confirmed. Topical agents such as 5-fluorouracil (5-FU), imiquimod, or diclofenac gel can target subclinical dysplastic changes in the surrounding skin, potentially reducing recurrence rates by treating the wider “field of cancerization”. In the context of this patient's recurrent corneal lesion, a combined approach, such as superficial shave excision followed by adjuvant cryotherapy or a course of topical chemotherapy might have offered a more definitive solution by addressing both the visible horn and its potential pathological base, while still aiming to preserve visual function and address cosmetic concerns.

When considering treatment strategies for Asian patients, specific factors such as skin type (Fitzpatrick III-V) and the higher propensity for post-inflammatory hyperpigmentation (PIH) must be taken into account (21, 22). Surgical excision is highly effective and remains a primary choice for ensuring complete pathological assessment; however, meticulous technique and post-operative care are crucial to minimize hypertrophic scarring and pigmentary changes. Among ablative therapies, CO2 laser has been reported to yield favorable cosmetic outcomes in Asian populations with cutaneous lesions, as it allows for precise control with minimal collateral damage, though the risk of PIH persists. Cryotherapy is also widely used, but its application should be cautious in pigmented skin due to the notable risk of hypopigmentation or hyperpigmentation. For superficial or clinically benign lesions, topical therapies like imiquimod or 5-fluorouracil can be effective alternatives that avoid scarring, making them suitable for cosmetically sensitive areas like the periocular region. Studies on actinic keratosis management in Asian cohorts suggest that a combination of lesion-directed therapy followed by field-directed therapy may provide optimal outcomes by addressing both visible lesions and subclinical damage while managing pigmentation risks. Therefore, in the context of this case, a tailored approach combining precise excision or laser ablation with adjunctive topical treatment could offer an optimal balance between efficacy and cosmesis for an Asian patient.

While conservative management (e.g., periodic shaving or curettage) was chosen in this case due to the patient's preference and initial benign histology, its drawbacks become particularly evident in cases of recurrence, as exemplified here. The primary limitation is its palliative rather than curative nature. By addressing only the superficial keratinous component and not the underlying pathological base, conservative approaches inherently carry a high risk of recurrence, leading to a cycle of repeated procedures. This not only increases cumulative healthcare costs and patient inconvenience but also elevates the long-term risk. Each recurrence and intervention poses potential trauma to the corneal surface, which could inadvertently compromise visual acuity over time, the very outcome the strategy aims to prevent. Furthermore, repeated sampling may not always capture a representative tissue specimen, potentially missing a focal area of malignant transformation within the lesion base. In contrast, more definitive modalities like surgical excision or laser ablation aim to eradicate the lesion entirely, thereby significantly reducing recurrence rates. Similarly, adjunctive pharmacological therapies can treat the surrounding field of subclinical damage. Therefore, for recurrent lesions, the short-term benefits of conservative management in preserving tissue must be weighed against the long-term risks of persistent regrowth, potential for missed malignancy, and cumulative procedural morbidity.

## 4 Strengthening novelty and mechanistic insights

To our knowledge, the failure of mechanical debridement alone necessitates a shift in management strategy. The choice of mechanical debridement as our primary intervention was based on several considerations: the patient's strong refusal of any invasive procedure, the benign histopathological findings, preservation of best-corrected visual acuity at 1.0, and the need to minimize corneal scarring risk. While this approach provided temporary cosmetic improvement and allowed for histological sampling, its limitations became evident through the rapid recurrence pattern, highlighting that debridement only addresses the superficial keratinized component without affecting the underlying pathological base.

However, given the patient's ongoing reluctance toward invasive treatments, we must carefully consider the feasibility of proposed novel therapies. While we present these as potential alternatives based on mechanistic reasoning, their use in this specific context would require careful risk-benefit assessment, particularly given the absence of histological malignancy. Photodynamic Therapy (PDT), topical field therapy, and immunomodulation are therefore not arbitrary alternatives but rational, mechanism-based strategies tailored to the specific biology inferred from this unique case.

### 4.1 Targeting UV-induced stem cell instability

The corneal location's inherent susceptibility to UV radiation, even from ambient exposure, suggests that limbal epithelial stem cells may harbor subclinical damage, driving the recurrent hyperkeratotic output. This hypothesis directly informs a two-pronged therapeutic strategy: using rigorous UV protection using UV-blocking contact lenses or protective eyewear is essential to mitigate continued insult (23, 24).

If recurrence continues, PDT with methyl aminolevulinate could be an ideal intervention. PDT is highly effective in treating actinic keratosis by selectively targeting dysplastic, UV-damaged cells. Its application could eradicate the clone of abnormal stem cells while sparing the surrounding healthy corneal tissue, thereby addressing the root cause beyond the visible keratin.

### 4.2 Reversing epigenetic dysregulation

The lesion's cyclical regeneration of histologically bland tissue suggests a persistently activated, pro-keratinization state in the local epithelium, potentially maintained by epigenetic mechanisms. This perspective shifts the therapeutic goal from physical removal to biochemical reprogramming.

This hypothesis provides a strong rationale for a trial of topical epigenetic-modifying agents. Topical 5-fluorouracil (5-FU) or diclofenac gel, which is standard treatments for cutaneous actinic keratosis, could be deployed here. They work by targeting rapidly proliferating cells and modulating the local inflammatory and dysplastic microenvironment, effectively “re-educating” the epithelial field and preventing regrowth (25, 26).

### 4.3 Restoring local immune surveillance

The non-inflammatory, non-bleeding base observed after each debridement implies a failure of the local immune system to recognize and eliminate the aberrant tissue. The goal here is to break this state of immune tolerance.

Topical imiquimod, an immune response modifier, represents a targeted approach. It acts by activating local toll-like receptors, stimulating an immune attack against the lesion that is otherwise absent (27, 28). Additionally, the application of a cryopreserved amniotic membrane following debridement could be beneficial. Beyond its structural role, the amniotic membrane possesses potent immunomodulatory and anti-inflammatory properties that can help reset the local ocular surface immune milieu, potentially preventing recurrence by restoring immune homeostasis.

### 4.4 Practical therapeutic considerations

It is crucial to acknowledge that while novel therapies like Hedgehog inhibitors or other targeted biological agents represent exciting research directions, their application to this benign corneal lesion is currently not justified (29). These agents carry substantial systemic side effects and are typically reserved for life-threatening malignancies. Furthermore, the absence of molecular targets and the lesion's benign nature make such approaches disproportionate to the clinical problem.

The optimal management strategy must balance several factors: the lesion's demonstrated benign histology, the patient's strong preference for minimal intervention, preservation of visual function, and the cosmetic concern that primarily motivates treatment. In this context, a staged approach beginning with the most conservative options, including enhanced UV protection, lubricants followed by carefully selected, minimally invasive interventions if absolutely necessary, represents the most prudent course. Future treatment decisions should be guided by documented progression or change in lesion characteristics rather than theoretical mechanisms alone.

## 5 Conclusion

This report documents a rare case of recurrent corneal-conjunctival keratosis with a tenacious recurrence pattern that belied its benign histology.

Recognizing the patient's limited adherence to intensive follow-up, we propose a simplified, patient-centric surveillance strategy that prioritizes feasibility and early detection of significant progression. The components include: (1) Monitoring, conducting slit-lamp examination and anterior segment photography only during the patient's self-initiated visits for cosmetic concerns or other ocular issues; (2) Focused Symptom Awareness, educating the patient to recognize and report key warning signs including rapid growth, color change to reddish hue, bleeding, or any visual disturbance; (3) Essential Protection, recommending consistent use of UV-protection eyewear during outdoor activities.

This streamlined approach replaces the previously proposed resource-intensive protocol with a more realistic framework that works within the patient's engagement level. By leveraging patient-initiated visits and emphasizing recognizable warning signs, this strategy maintains clinical vigilance without imposing undue burden. This case highlights that for benign but recurrent ocular surface lesions, establishing a practical surveillance plan aligned with patient behavior is crucial for long-term management, even when it falls short of ideal monitoring standards.

## 6 Limitation

We hypothesize that this lesion may represent an early or variant form of AK, though this remains speculative without supporting molecular data. The potential etiological roles of UV exposure, HPV infection, or epigenetic dysregulation discussed below are theoretical considerations based on the literature rather than evidence-based conclusions from this case.

A key limitation of this report is the lack of molecular investigation. Tests such as HPV PCR or immunohistochemical staining for p53 and Ki-67 were considered but not performed due to the scant debrided material and the patient's refusal of additional invasive procedures.

## Data Availability

The original contributions presented in the study are included in the article/supplementary material, further inquiries can be directed to the corresponding author.
